# The influence of adipose-derived stromal vascular fraction cells on the treatment of knee osteoarthritis

**DOI:** 10.1186/s12891-020-03231-3

**Published:** 2020-04-06

**Authors:** Masanori Tsubosaka, Tomoyuki Matsumoto, Satoshi Sobajima, Takehiko Matsushita, Hideki Iwaguro, Ryosuke Kuroda

**Affiliations:** 1 Department of Orthopaedic Surgery, Kobe University Graduate School of Medicine, Kobe, Japan; 2 Department of Orthopaedic Surgery, Sobajima Clinic, 2-2-6, Aramotokita, Higashiosaka, Osaka, 577-0011 Japan

**Keywords:** Adipose-derived stromal vascular fraction cells, Stem cell, Osteoarthritis, T2 mapping

## Abstract

**Background:**

Adipose-derived stromal vascular fraction (SVF) cells are a mixed cell population that includes cells with multilineage potential, similar to bone marrow-derived mesenchymal stem cells. Our purpose is to investigate the influence of SVF cells in patients with knee osteoarthritis (OA) and the short-term treatment effects.

**Methods:**

Fifty-seven patients were enrolled and treated with intra-articular injection of 2.5 × 10^7^ SVF cells into the knee joint between September 2017 and March 2018. All patients were followed up for 12 months or longer. Mean age at treatment and follow-up period were 69.4 ± 6.9 years and 13.7 ± 2.0 months, respectively. The mean preoperative hip-knee-ankle angle was 6.7 ± 3.6°. SVF cells were prepared using the Celution®800/CRS system from the patients’ abdominal or breech subcutaneous fat. The mean SVF cell viability was 90.6 ± 2.7%. Clinical evaluations were performed for range of motion, Western Ontario and McMaster Universities Osteoarthritis Index (WOMAC), visual analog scale (VAS) for pain, and the Knee injury and Osteoarthritis Score (KOOS). Imaging evaluations, which included the hip-knee-ankle angle assessed via radiography, and T2 mapping value using a 1.5-T magnetic resonance imaging unit were also assessed. Both clinical and imaging evaluations were performed preoperatively, 1, 3, 6, and 12 months postoperatively, and compared among all timepoints (*p* < 0.05).

**Results:**

Knee extension angle at 6 and 12 months postoperatively was significantly better than the preoperative angle. Total WOMAC, VAS, and KOOS scores at 1, 3, 6 and 12 months postoperatively were significantly better than preoperative scores. There was no significant difference in hip-knee-ankle angle among the five time periods. T2 mapping values of lateral femur and tibia were significantly higher 12 months postoperatively than preoperatively.

**Conclusions:**

The short-term clinical effects of intra-articular SVF cell injection on knee OA were excellent. Intra-articular SVF cell injection is a novel and innovative approach for treating patients with knee OA.

## Background

Osteoarthritis (OA), a chronic degenerative joint disorder characterized by articular cartilage destruction and osteophyte formation, is a prevalent cause of significant disability. Disability is particularly evident in the elderly, where 10–50% of the senior population is affected by OA and many are severely disabled [1, 2]. Knee OA initiates changes in the cartilage, ligaments, tendons, and muscles of the knee joint [3], which lead to knee buckling, poor psychosocial outcomes, increased risk of falls, balance deficits, and limitation in certain physical activities [4–6]. The altered clinical status and functional disability lead to a decrease in the quality of life [7]. Recently, cell therapy with adipose tissue-derived mesenchymal stem cells (ADSCs) is attracting attention as a novel potential therapy for knee OA [8, 9]. ADSCs share similar properties with bone marrow-derived mesenchymal stem cells (BMSCs), but they are easier to collect for clinical application, with higher isolation yields [9]. ADSCs, however, require culturing, and it takes a few weeks between isolation and application.

Adipose-derived stromal vascular fraction (SVF) cells contain regenerative cells, such as ADSCs, macrophages, blood cells, pericytes, fibroblasts, vessel-forming cells like endothelial and smooth muscle cells, and their progenitors [10–12]. SVF cells can be easily isolated in large amounts from autologous adipose tissue and used without culturing or differentiation [13, 14]. The safety and efficacy of SVF cells have been examined in several clinical settings, such as cardiology [15], urology [16], plastic, and reconstructive surgery [17, 18]. Studies have also reported the effectiveness of SVF cells in orthopedic clinical settings [19–21]. However, the detailed clinical evaluation of SVF cell treatment for knee OA while securing the sample size has not yet been reported in a large number of patients.

Based on this scientific background, we report a prospective case series of intra-articular injection of autologous SVF cells in knee OA. We investigated the short-term treatment effects in detail, and evaluated the safety, feasibility, and efficacy of intra-articular injections of autologous SVF cells.

## Methods

### Study design and criteria for subject enrollment

This clinical study was designed to evaluate the safety, feasibility, and efficacy of autologous SVF cells in patients with knee OA. The grade of knee OA was evaluated by the Kellgren-Lawrence (KL) classification, and all patients with grades I to IV OA participated in this study. The study protocol conformed to the Declaration of Helsinki and was approved by the appropriate ethics committees. All patients provided informed consent prior to participation.

The inclusion criteria were (a) patients diagnosed with knee OA at any age, (b) exhibiting substantial pain and loss of function, (c) ineffectiveness of conservative treatment including rehabilitation, medication, and intra-articular injection of hyaluronic acid or steroids, and (d) written informed consent. The exclusion criteria were (a) severe bony defect seen on preoperative radiographs, (b) previous knee injury requiring operation, (c) active or previous knee joint infection, and (d) a serious past history, such as systemic inflammatory diseases and vascular changes.

Patients were asked to perform daily home exercises by themselves according to a standardized rehabilitation protocol of the hospital after treatment, in addition to rehabilitation by a physical therapist through regular hospital visits.

### Treatment procedures (Fig. 1)

The Celution® 800/CRS system (Cytori Therapeutics Inc., San Diego, CA) was used to extract SVF cells from the patient’s abdominal or breech subcutaneous fat. This system consists of two parts: one for tissue washing and digestion, and the other for cell concentration. All subjects underwent a liposuction procedure to obtain 100–360 mL of adipose tissue under general anesthesia; the extracted tissue was then processed using the Celution® 800/CRS System according to manufacturer instructions. Briefly, the tissue was washed to remove blood and debris. Celase® GMP, which was a mixture of highly purified collagenase and neutral protease enzymes, was then added and incubated at ~ 37 °C for 20 min with continuous mixing to digest the aspirated adipose tissue. After digestion, the SVF cells were concentrated using centrifugation and washed to remove the Celase® reagent. SVF cells were then extracted from the system and counted to prepare the specified dose in 5 mL of lactated Ringer’s solution. The whole system can be operated aseptically using clinical-grade solutions such as saline and lactated Ringer’s, and single-use Celution™ consumable sets. The SVF cell count and viability were determined at each investigational site using the NC-100™ NucleoCounter® Automated Cell Counting System (Chemometec, Allerod, Denmark). This system is an image cytometer based on fluorescence from the fluorescent dye, propidium iodide. When a sample is mixed with Reagent A100 and Reagent B, lysis of the viable cell membrane occurs, rendering all the cell nuclei susceptible to staining with propidium iodide facillitating in a total cell count. However, non-viable cells are permeable without treatment and are therefore stained directly with propidium iodide, resulting in a non-viable cell count. Thus, the cell viability of a sample is determined using the total cell count and the count of non-viable cells.

**Fig. 1:**
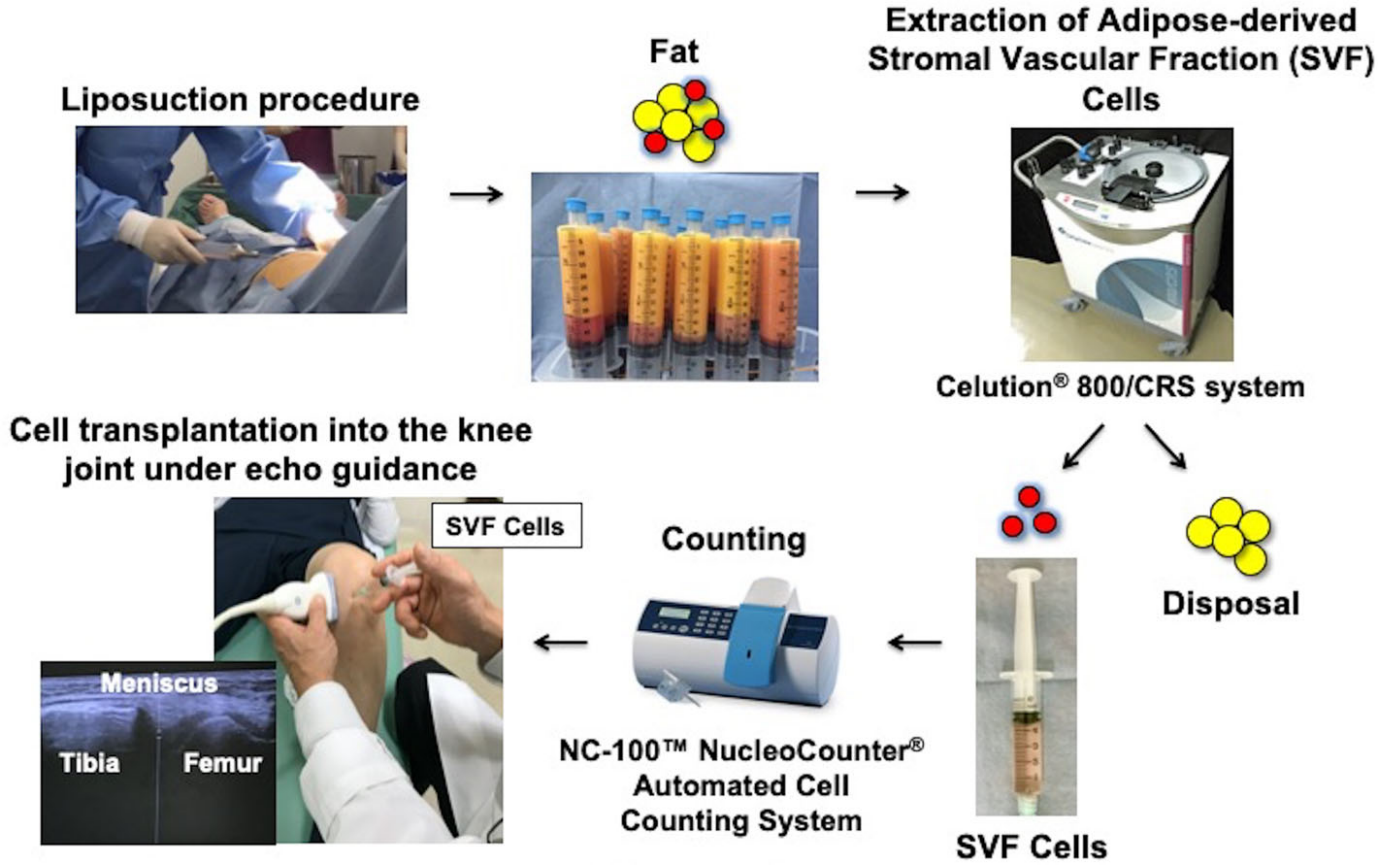
Schema of treatment procedures

We administered an intra-articular injection of 2.5 × 10^7^ SVF cells to each patient according to the number of purified SVF cells and guidelines previously stated in a similar report [22]. Cell transplantation into the knee joint was performed without anesthetic and under echo guidance after aspiration if the joint fluid level was excessive.

### Endpoints

The primary endpoint of this study was patient improvement based on clinical evaluations and scores. Clinical evaluations included knee range of motion (ROM) and muscle force of knee extension and flexion using a hand-held dynamometer. Clinical scores included Western Ontario and McMaster Universities Osteoarthritis Index (WOMAC), visual analog scale (VAS) for pain (0–100), Japanese Knee Osteoarthritis Measure (JKOM), and Knee injury and Osteoarthritis Outcome Score (KOOS). For measurement of muscle force of knee extension and flexion, patients were tested in a prone position with their knee at 45 degrees flexion. The hand-held dynamometer was placed at the center of their lower leg. The examiner asked subjects to bend their knee and hold for 3 s to measure hamstrings strength, and to straighten their knee and hold for 3 s to measure quadriceps strength. The examiner added resistance to maintain the knee at 45° and measured the displayed value as muscle strength. These tests were performed three times and the average value was recorded.

**Fig. 2:**
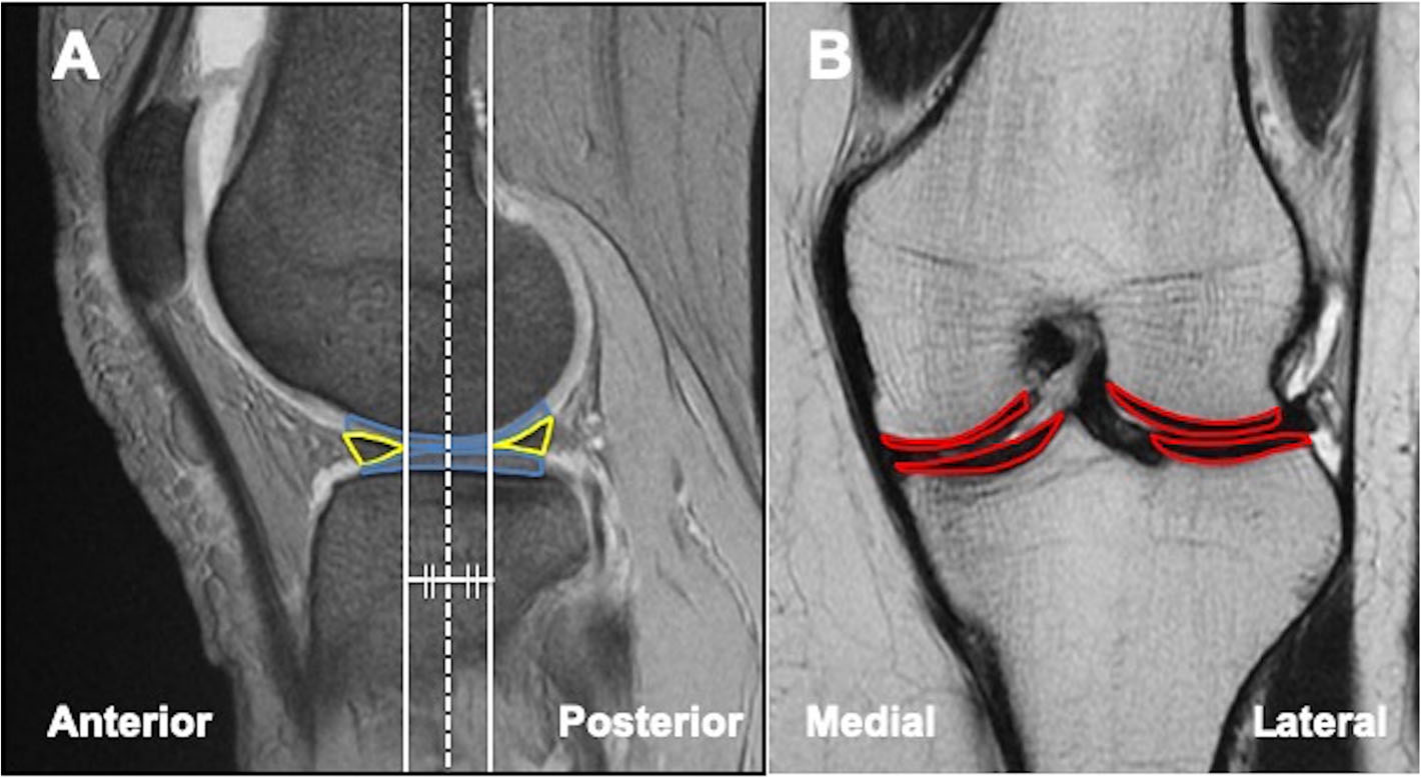
Method of calculating the T2 mapping value. **a** In a sagittal slice of T1-weighted fast-field echo images, we select a central slice (white dotted line) that passed through the center of the weightbearing cartilage (blue line) surrounded by the anterior and posterior margins of the meniscus (yellow line). In addition to the central slice, we added two slices neighboring the central slice anteriorly and posteriorly. **b** The region of interest (ROI) was set at the weight-bearing full-thickness cartilage (red line) of the medial and lateral femoral condyle and medial and lateral tibial plateau on the central slice of the coronal image. The ROI was also set using the same method on both the anterior and posterior slice. Overall, the T2 mapping values of 12 ROIs were measured

As a secondary endpoint, imaging evaluations, which included the hip-knee-ankle (HKA) angle assessed via radiography, and T2 mapping value using a 1.5-T magnetic resonance imaging (MRI) unit (Sigma Exite HDx; GE Healthcare, Waukesha, Wis) [23, 24] were also assessed. The method of calculating the T2 mapping value is as follows; we selected a central slice that passed through the center of the weight-bearing cartilage surrounded by the anterior and posterior margins of the meniscus on a sagittal slice of T1-weighted fast-field echo images. In addition to the central slice, we added two slices neighboring the central slice anteriorly and posteriorly (Fig. 2a). The region of interest (ROI) was then set at the weight-bearing full-thickness cartilage of the medial and lateral femoral condyle and medial and lateral tibial plateau on the central slice of the coronal image (Fig. 2b). The ROI was also set using the same method on both the anterior and posterior slice. Overall, the T2 mapping values of 12 ROIs were measured. According to this analysis, the lower the T2 mapping value, the lower the degree of articular cartilage degeneration.

Both clinical and imaging evaluations were performed preoperatively and at 1, 3, 6 and 12 months postoperatively after intra-articular injection of SVF cells. Clinical evaluations were performed by an independent experienced physiotherapist. Image analyses were performed by an independent orthopedic surgeon with 15 years of experience in MRI analysis of knee OA features. For safety evaluations, incidence, severity, and outcome of all adverse events were recorded.

### Statistical analysis

All values were expressed as mean ± standard deviation. Results were analyzed using a statistical software package (Statview 5.0; Abacus Concepts, Inc., Berkeley, CA, USA). Clinical and imaging evaluations were compared between the five time periods using repeated measures analysis of variance. Furthermore, we evaluated the clinical scores preoperatively and at 12 months postoperatively, and investigated the improvement rate of clinical scores from preoperatively to 12 months postoperatively among the KL classification by using repeated measures analysis of variance. *P* < 0.05 was considered statistically significant. A statistical power analysis was performed prior to the study, which was expected to require a power of 0.8, based on a prespecified significance level of α < 0.05 and assuming a medium effect size (effect size = 0.30) using G power 3 [25]. The estimated sample size was 45 patients.

## Results

**Fig. 3:**
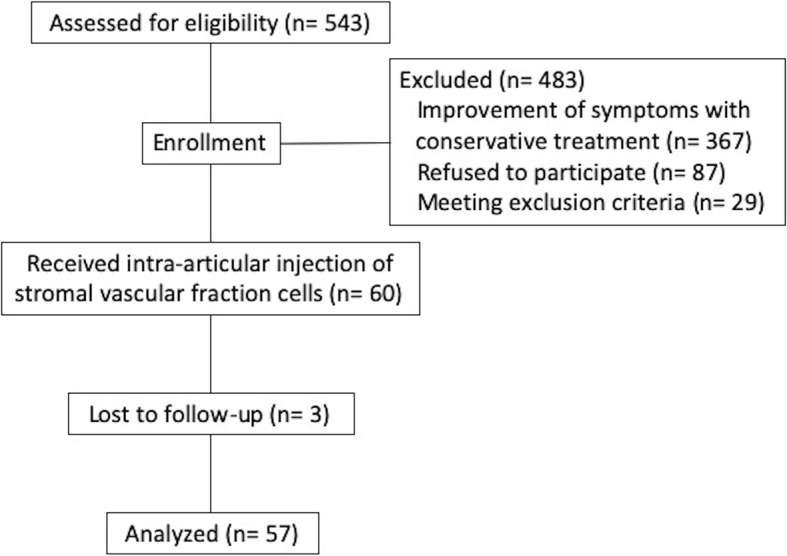
Patients flow diagram

In total, 543 patients visited our clinic for SVF cell treatment between September 2017 and March 2018. Of them, 367 were excluded because they showed improvements in symptoms with conservative treatment including rehabilitation, medication, and intra-articular injection of hyaluronic acid or steroids. Eighty-seven patients refused to participate in this study. Twenty-nine were excluded based on exclusion criteria: (a) patients with severe bony defect observed on preoperative radiographs (7 patients), (b) patients with previous knee injury requiring operation (2 patients), (c) patients with active or previous knee joint infection (1 patients), and (d) patients with a serious past history (19 patients). As a result, 60 patients were enrolled and treated with intra-articular injection of SVF cells into the knee joint. Three patients were lost to follow-up, leaving a total of 57 patients (57 knees) available for the study. The follow-up rate was 95.0%. (Fig. 3). All patients were followed up for 12 months or longer. Mean age at treatment, follow-up period, and body mass index were 69.4 ± 6.9 years, 13.7 ± 2.0 months, and 25.1 ± 3.1 kg/m2, respectively. Patients were divided based on the KL classification: grade I, 0 patients; grade II, 11 patients; grade III, 36 patients; and grade IV, 10 patients. The mean preoperative HKA angle was 6.7 ± 3.6° (varus type knee OA, 53 knees; valgus knee OA, 4 knees), and the mean preoperative knee extension and flexion angles were − 6.0 ± 5.9° and 131.3 ± 14.2°, respectively (Table 1).

**Table 1:** Patient characteristics

Characteristics	Baseline Data
Sex (M/F); n (%)	41/16 (72%/28%)
Age (mean ± standard deviation); yrs	69.4 ± 6.9
Body mass index; kg/m^2^	25.1 ± 3.1
Duration of follow-up; months	13.7 ± 2.0
Hip-knee-ankle angle at baseline; degree	6.7 ± 3.6
Knee extension angle; degree	− 6.0 ± 5.9
Knee flexion angle; degree	131.3 ± 14.2
Kellgren- Lawrence classificationn (%)	
I	0 (0%)
II	11 (19%)
III	36 (63%)
IV	10 (18%)

The mean volume of liposuction and number of purified SVF cells were 334.3 ± 44.0 mL and 7.6 × 10^7^ ± 2.5 × 10^7^, respectively. Mean SVF cell viability was 90.6% ± 2.7%.

### Clinical evaluation

The mean ROM improved from a baseline of − 6.0°–131.3° to − 4.8°–133.9° at 1 month, − 4.3°–134.3° at 3 months, − 3.7°–134.5° at 6 months, and − 3.5°–132.6° at 12 months postoperatively. The improvement in the mean extension angle from baseline to 6 and 12 months was statistically significant. Muscle force of knee extension and flexion improved from a baseline of 202.5 Nm and 99.5 Nm, respectively, to 198.9 Nm and 108.2 Nm at 1 month, 219.0 Nm and 116.6 Nm at 3 months, 235.4 Nm and 124.2 Nm at 6 months, and 261.9 Nm and 126.8 Nm at 12 months postoperatively, respectively. The mean muscle force of knee extension and flexion was significantly better at 12 months postoperatively than preoperatively.

The improvement in the mean total WOMAC scores from baseline to 12 months postoperatively was from 33.4 to 22.6 points, which showed a statistically and clinically significant difference (Table 2). The improvement in VAS scores from baseline to 12 months postoperatively was from 46.5 to 32.8 points, which showed a statistically and clinically significant difference (Table 2). An improvement was also evident in the mean total JKOM scores from baseline to 12 months postoperatively (from 34.9 to 26.8 points), which showed a statistically significant difference (Table 2). Improvements in the KOOS score were also evident, including an average score of 5 subscales and all subscale scores 12 months postoperatively (Table 2, Additional file 1). The improvements from baseline in the mean scores of pain, symptoms, activities of daily living, sports, and quality of life subscales were from 53.1 to 66.3 points, 57.8 to 67.0 points, 70.0 to 77.5 points, 27.6 to 37.5 points, and 33.6 to 44.6 points with statistical significance, respectively. The improvements in the mean scores of pain, symptoms, sports, and quality of life subscales also showed clinically significant differences (Additional file 1).

**Table 2:** Clinical evaluation results

Range of motion of the knee					
Extension	**Mean value ± S.D. (°)**	***P*value**	**Flexion**	**Mean value ± S.D. (°)**	***P*value**
Preoperative	−6.0 ± 5.9		Preoperative	131.3 ± 14.2	
1 month	−4.8 ± 4.8	0.23	1 month	133.9 ± 12.5	0.37
3 months	−4.3 ± 4.5	0.10	3 months	134.3 ± 11.9	0.29
6 months	−3.7 ± 4.4	0.02^a^	6 months	134.5 ± 12.4	0.26
12 months	−3.5 ± 4.1	0.02^a^	12 months	132.6 ± 15.2	0.67
Muscle force					
Extension (Quadriceps)	**Mean value ± S.D. (Nm)**	***P*value**	**Flexion (Hamstrings)**	**Mean value ± S.D. (Nm)**	***P*value**
Preoperative	202.5 ± 85.9		Preoperative	99.5 ± 39.7	
1 month	198.9 ± 91.1	0.86	1 month	108.2 ± 41.3	0.36
3 months	219.0 ± 103.8	0.43	3 months	116.6 ± 59.8	0.07
6 months	235.4 ± 104.5	0.11	6 months	124.2 ± 41.7	< 0.01^a^
12 months	261.9 ± 106.7	< 0.01^a^	12 months	126.8 ± 38.7	< 0.01^a^
Western Ontario and McMaster Universities Osteoarthritis Index			**Visual analog scale**		
Total Score	**Mean value ± S.D.**	***P*value**		**Mean value ± S.D.**	***P*value**
Preoperative	33.4 ± 18.2		Preoperative	46.5 ± 23.5	
1 month	26.3 ± 14.6	0.046^a^	1 month	30.1 ± 18.8	< 0.01^a^
3 months	22.8 ± 15.7	< 0.01^a^	3 months	27.3 ± 17.6	< 0.01^a^
6 months	22.6 ± 16.4	< 0.01^a^	6 months	27.4 ± 18.8	< 0.01^a^
12 months	22.6 ± 17.5	< 0.01^a^	12 months	32.8 ± 24.7	< 0.01^a^
Japanese Knee Osteoarthritis Measure			**Knee injury and Osteoarthritis Outcome Score**		
Total Score	**Mean value ± S.D.**	***P*value**	**Average Score of 5 subscales**	**Mean value ± S.D.**	***P*value**
Preoperative	34.9 ± 18.2		Preoperative	48.7 ± 15.8	
1 month	30.5 ± 17.1	0.26	1 month	55.2 ± 17.6	0.04^a^
3 months	25.8 ± 17.6	0.02^a^	3 months	58.6 ± 15.4	< 0.01^a^
6 months	24.5 ± 17.8	< 0.01^a^	6 months	59.2 ± 15.8	< 0.01^a^
12 months	26.8 ± 19.7	0.04^a^	12 months	58.6 ± 16.8	< 0.01^a^

^a^ Statistically significant, Standard deviation (S.D.)

According to the KL classification, there were no grade I patients in this study. There was no significant difference in preoperative clinical scores of WOMAC, VAS, JKOM, and KOOS among the KL classifications. Clinical scores of WOMAC, JKOM and KOOS at 12 months postoperatively were significantly better for grade II than for grade III. Furthermore, clinical scores of WOMAC, VAS, and JKOM at 12 months postoperatively were also significantly better for grade II than for grade IV. The improvement rate of WOMAC from baseline to 12 months was significantly better for grade II than for grade III. There was no significant difference in improvement rates of VAS, JKOM, and KOOS among the KL classifications (Table 3).

**Table 3:** Improvement rate from baseline to 12-month postoperatively in Western Ontario and McMaster Universities Osteoarthritis Index (WOMAC), visual analog scale (VAS) for pain, Japanese Knee Osteoarthritis Measure (JKOM), and Knee injury and Osteoarthritis Outcome Score (KOOS) scores among Kellgren-Lawrence classifications

	Clinical score	Kellgren-Lawrence classification			*P* value		
		Grade II (11 patients)	Grade III (36 patients)	Grade IV (10 patients)	GradeII vs III	GradeII vs IV	GradeIII vs IV
Preoperative	WOMAC	27.7 ± 15.1	35.1 ± 17.5	37.6 ± 21.5	0.27	0.21	0.70
score	VAS	35.5 ± 18.6	52.1 ± 22.7	55.4 ± 30.8	0.07	0.06	0.70
	JKOM	27.4 ± 15.3	37.4 ± 18.5	37.1 ± 20.8	0.15	0.24	0.97
	KOOS	52.9 ± 12.0	48.6 ± 17.5	45.4 ± 14.6	0.46	0.28	0.56
12 months	WOMAC	11.2 ± 9.4	26.0 ± 16.6	25.7 ± 17.1	0.01^a^	0.04^a^	0.96
score	VAS	20.7 ± 20.2	37.4 ± 24.6	42.4 ± 25.8	0.07	0.045^a^	0.56
	JKOM	14.4 ± 9.6	31.6 ± 20.1	32.2 ± 18.5	0.01^a^	0.03^a^	0.92
	KOOS	68.5 ± 14.3	56.3 ± 17.9	56.1 ± 14.0	0.048^a^	0.08	0.97
Improvement	WOMAC	58.5 ± 26.5	22.8 ± 46.8	28.8 ± 22.1	0.02^a^	0.09	0.67
rate	VAS	14.8 ± 20.0	14.7 ± 20.9	13.0 ± 18.3	0.99	0.84	0.81
	JKOM	34.4 ± 49.9	7.4 ± 56.6	5.7 ± 27.8	0.15	0.20	0.92
	KOOS	19.1 ± 24.2	11.2 ± 27.9	17.7 ± 21.2	0.41	0.90	0.47

Mean value ± Standard deviation (S.D.) ^a^ Statistically significant

### Imaging evaluation

**Fig. 4:**
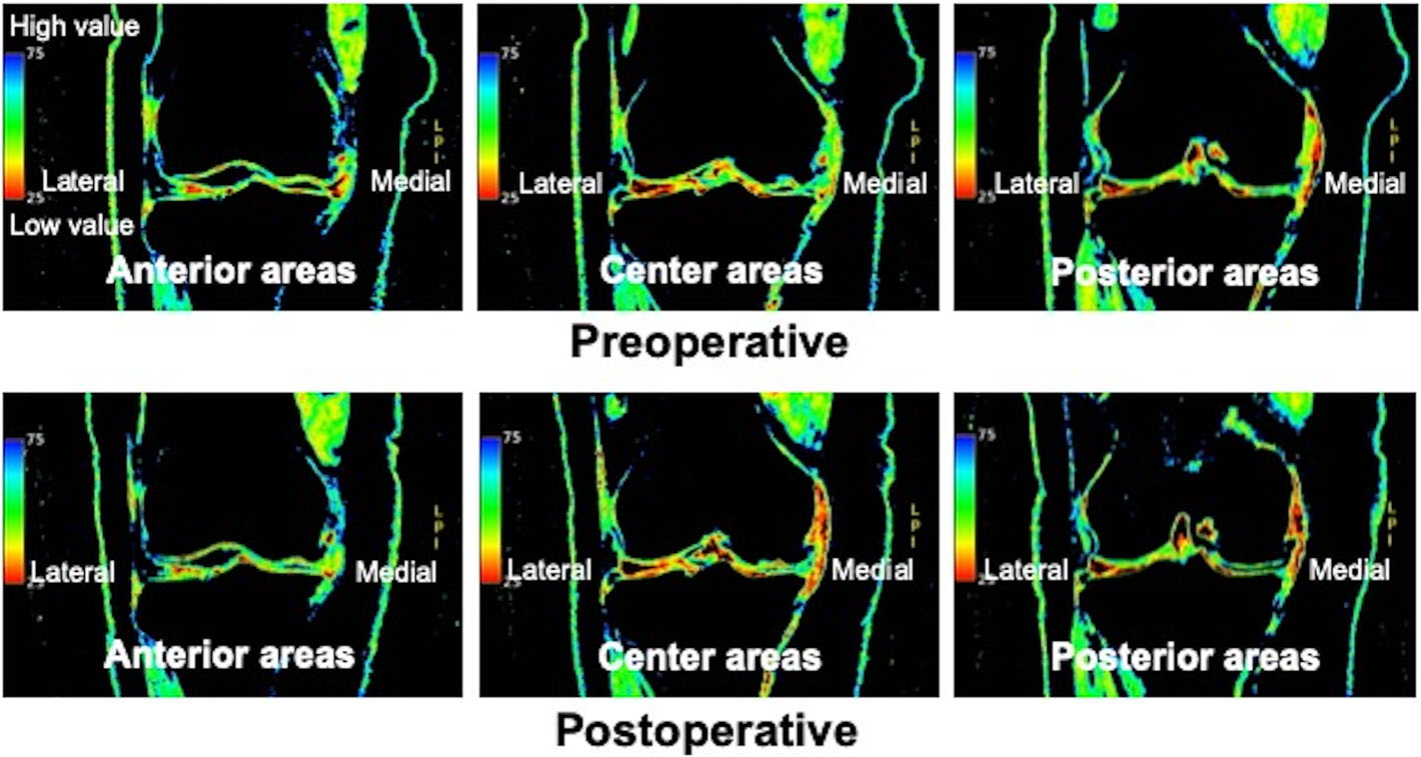
An example in which the mean T2 mapping values were significantly lower than those preoperatively

The mean HKA angles changed from a baseline of 6.7° to 7.1° at 1 month, 6.7° at 3 months, 6.6° at 6 months, and 6.8° at 12 months postoperatively. However, this change was not statistically significant at any time. In contrast, the mean T2 mapping values of the lateral femur and tibia in the anterior areas at 12 months postoperatively were significantly lower than those preoperatively. The mean T2 mapping value of the medial tibia in the central area at 6 and 12 months postoperatively was also significantly better than that preoperatively. Furthermore, the mean T2 mapping value of the lateral femur in the posterior area at 12 months postoperatively was significantly better than that preoperatively (Table 4, Fig. 4).

**Table 4:** Imaging evaluation results

Hip-knee-ankle angle	Mean value ± S.D.	*P* value						
Preoperative	6.7 ± 3.6							
1 month	7.1 ± 3.5	0.59						
3 months	6.7 ± 3.9	0.94						
6 months	6.6 ± 3.6	0.92						
12 months	6.8 ± 3.7	0.90						
Anterior T2 mapping value			**Central T2 mapping value**			**Posterior T2 mapping value**		
Medial femur	**Mean value ± S.D.**	*P***value**	**Medial femur**	**Mean value ± S.D.**	*P***value**	**Medial femur**	**Mean value ± S.D.**	*P***value**
Preoperative	51.5 ± 5.0		Preoperative	51.4 ± 4.6		Preoperative	52.9 ± 5.2	
1 month	50.0 ± 2.4	0.20	1 month	50.7 ± 4.1	0.60	1 month	51.2 ± 4.8	0.25
3 months	50.7 ± 4.1	0.52	3 months	50.7 ± 4.2	0.62	3 months	51.6 ± 4.8	0.37
6 months	50.1 ± 3.0	0.22	6 months	49.8 ± 4.7	0.28	6 months	52.0 ± 4.4	0.56
12 months	50.3 ± 4.6	0.054	12 months	50.4 ± 5.4	0.18	12 months	51.8 ± 4.8	0.22
Medial tibia			**Medial tibia**			**Medial tibia**		
Preoperative	42.6 ± 7.2		Preoperative	42.0 ± 6.0		Preoperative	42.1 ± 6.7	
1 month	40.6 ± 6.0	0.26	1 month	40.7 ± 5.0	0.39	1 month	40.7 ± 5.4	0.39
3 months	40.8 ± 5.5	0.29	3 months	40.4 ± 5.8	0.28	3 months	41.0 ± 5.8	0.52
6 months	39.1 ± 3.3	0.053	6 months	38.4 ± 2.7	0.02^a^	6 months	39.3 ± 3.2	0.11
12 months	39.7 ± 3.9	0.12	12 months	37.9 ± 2.8	0.01^a^	12 months	38.8 ± 3.8	0.08
Lateral femur			**Lateral femur**			**Lateral femur**		
Preoperative	43.4 ± 3.3		Preoperative	48.1 ± 4.7		Preoperative	47.0 ± 4.7	
1 month	42.3 ± 3.7	0.24	1 month	47.9 ± 4.8	0.85	1 month	46.1 ± 4.6	0.49
3 months	42.4 ± 3.2	0.29	3 months	46.7 ± 3.8	0.27	3 months	45.1 ± 3.3	0.13
6 months	41.8 ± 3.4	0.10	6 months	47.3 ± 4.0	0.56	6 months	44.6 ± 4.4	0.07
12 months	41.3 ± 1.7	0.047^a^	12 months	46.4 ± 2.4	0.22	12 months	43.4 ± 3.1	0.01^a^
Lateral tibia			**Lateral tibia**			**Lateral tibia**		
Preoperative	39.6 ± 2.1		Preoperative	37.2 ± 2.5		Preoperative	37.4 ± 2.5	
1 month	38.3 ± 2.2	0.06	1 month	36.6 ± 3.5	0.43	1 month	37.3 ± 2.9	0.85
3 months	39.3 ± 2.8	0.72	3 months	36.9 ± 2.3	0.67	3 months	38.0 ± 2.9	0.45
6 months	37.7 ± 2.3	0.01^a^	6 months	35.7 ± 2.4	0.07	6 months	37.1 ± 2.6	0.67
12 months	37.6 ± 1.5	0.04^a^	12 months	36.7 ± 2.2	0.60	12 months	37.1 ± 2.6	0.74

^a^ Statistically significant, Standard deviation (S.D)

### Safety evaluation

Neither deaths nor life-threatening adverse events were observed during the 12-month follow-up after cell therapy. Furthermore, there was no mild to moderate adverse event such as swelling, local heat of the knee, or infection during follow-up.

## Discussion

Clinical evaluation showed widespread improvement in multiple parameters early after intra-articular SVF cell injection into the knees of OA patients. Most imaging evaluations, especially T2 mapping values, showed similar trends; however, most of these imaging evaluations did not achieve statistical significance. This may be because of the well-known placebo effect of injectable therapies in patient-reported outcomes, or to the limited follow-up duration, as most studies only evaluate the knee via MRI over the course of 1–2 years.

There have been a few reports on good clinical results of ADSC cell therapy for knee OA [8, 9]. ADSCs and BMSCs share similar properties, but require culturing after isolation. In contrast, SVF cells are not cultured and can be prepared from and re-injected back into the patient within the same procedure. Equivalent to BMSCs, SVF cells contain cells with multilineage potential and can be easily isolated in large amounts from autologous adipose tissues and used without culturing [13, 14]. SVF cells have been used for various clinical purposes [15–18], and studies on autologous SVF cells for the treatment of knee OA have been reported [19–21]. Fodor et al. reported that autologous adipose derived SVF cells were safe and presented a new potential therapy for pain reduction in knee OA, and Hong et al. reported that SVF cell treatment could be more effective than treatment with hyaluronic acid, although their sample size was small [19]. Although Michialek et al. reported that a large clinical trial of intra-articular SVF cell injections were a safe and clinically effective strategy leading to improved quality of life, detailed clinical evaluations were not performed [20]. In this clinical study, we performed the detailed clinical evaluation while securing the sample size.

In the current investigation, the mean total WOMAC, JKOM, VAS, and average 5-subscale KOOS scores 3, 6, and 12 months postoperatively were significantly better than preoperative scores. This was particularly evident in WOMAC and KOOS, for which all subscales at 3, 6, and 12 months postoperatively were significantly better than those preoperatively. The WOMAC instrument is a 24-item patient-reported instrument developed to assess pain, stiffness, and physical functioning in patients with hip or knee OA [26]. The physical function section of the WOMAC provides patients with a list of daily activities and requires them to state how difficult the activities were in the last 48 h because of their arthritis. KOOS is a disease-specific, patient-reported outcome measure assessing perceived pain, other symptoms, ADL, sports and recreation functions, and knee-related quality of life. It is freely accessible and intended for use in the short and long term for research and clinical purposes [27]. WOMAC physical function and KOOS ADL items are identical. Sports and recreation functions and knee-related quality of life subscales were not referred in the WOMAC. Intra-articular SVF cell injection into knees with OA was thought to greatly improve sporting activities that required a higher level of activity than ADL, as well as knee-related quality of life.

T2 mapping is a quantitative cartilage imaging technique that facilitates detection of changes in water and collagen content. Thus, T2 mapping values reflect the degree of articular cartilage degeneration [23, 24]. Although obvious improvement in coronal alignment was not observed in this study, the mean T2 mapping values of the lateral tibia in the anterior area and lateral femur in the anterior and posterior areas at 12 months postoperatively were significantly lower than those seen preoperatively. This indicated that the extent of articular cartilage degeneration was improved even if no obvious structural change was observed via MRI. Furthermore, the T2 mapping value of the lateral femur and tibia confirmed this improvement because mechanical stress was not applied on the lateral side, and almost all patients included in this study (55 of 59 patients; 93.2%) had varus knee OA. The mean T2 mapping value of the medial tibia in the central area at 6 and 12 months postoperatively was also significantly lower than those seen preoperatively. This result was thought to be due to the fact that the region of interest was set in the remaining cartilage at the non-weighted part, because cartilage defect was found in the weighted part of the medial tibia in almost all the cases in this study.

We evaluated the clinical scores preoperatively and 12 months postoperatively and investigated the improvement rate of clinical scores from preoperatively to 12 months postoperatively among the KL classification. The improvement rate of WOMAC scores from baseline to 12 months was significantly better for grade II than for grade III and tended to be better for grade II than for grade IV. Furthermore, the improvement rate of JKOM scores tended to be better for grade II than for grade III and IV. These results indicated that it was desirable to perform the treatment of SVF cells before the degree of degeneration of knee OA had progressed excessively.

This study has some limitations. First, this study had no control group. We plan to investigate the association between SVF cells and other intra-articular interventions in the future. Second, clinical and imaging evaluations were only performed preoperatively, and at 1, 3, 6, and 12 months after intra-articular SVF cell injection into the knee. Long-term investigation of clinical and structural changes is now ongoing. Third, we did not evaluate the relationship between dosage of intra-articular SVF cell injection and clinical/structural results. Finally, this study applied a single treatment of SVF cells. Optimal treatment may require multiple injections.

## Conclusions

We performed detailed clinical evaluations of intra-articular autologous SVF cell injection for knee OA while securing the sample size, and obtained good short-term clinical results. All procedures were performed safely. The short-term clinical evaluation of intra-articular SVF cell injection on knee OA was very promising. We suggest intra-articular SVF cell injection into the knee joint as an innovative approach to treat patients with knee OA.

## Supplementary information

**Additional file 1.** Each subscale score of Western Ontario and McMaster Universities Osteoarthritis Index, visual analog scale, Japanese Knee Osteoarthritis Measure, and Knee injury and Osteoarthritis Score.

## Data Availability

The datasets used and/or analyzed during the current study are available from the corresponding author on reasonable request.

## Abbreviations

OA: Osteoarthritis
ADSCs: Adipose tissue-derived mesenchymal stem cells
BMSCs: Bone marrow-derived mesenchymal stem cells
SVF: Stromal vascular fraction
KL: Kellgren-Lawrence
ROM: range of motion
WOMAC: Western Ontario and McMaster Universities Osteoarthritis Index
VAS: Visual analog scale
JKOM: Japanese Knee Osteoarthritis Measure
KOOS: Knee injury and Osteoarthritis Outcome Score
HKA: Hip-knee-ankle
MRI: Magnetic resonance imaging
ADL: Activities of daily living
