# Supplementary material for: The influence of adipose-derived stromal vascular fraction cells on the treatment of knee osteoarthritis
Source: BMC Musculoskelet Disord. 2020 Apr 6;21:207. doi: 10.1186/s12891-020-03231-3 (PMC7137313; doi:10.1186/s12891-020-03231-3)
Supplement: Supplementary file 1 — Additional file 1. Each subscale score of Western Ontario and McMaster Universities Osteoarthritis Index, visual analog scale, Japanese Knee Osteoarthritis Measure, and Knee injury and Osteoarthritis Score. [file 12891_2020_3231_MOESM1_ESM.docx]

**Additional file 1.** Each subscale score of Western Ontario and McMaster Universities Osteoarthritis Index, visual analog scale, Japanese Knee Osteoarthritis Measure, and Knee injury and Osteoarthritis Outcome Score.

| Western Ontario and McMaster Universities Osteoarthritis Index | | | | | | | | |
| --- | --- | --- | --- | --- | --- | --- | --- | --- |
| Total Score | **Mean value ± S.D.** | | **P value** | **Pain subscale** | **Mean value ± S.D.** | | **P value** | |
| Preoperative | 33.4 ± 18.2 | |  | Preoperative | 12.9 ± 7.0 | |  | |
| 1 month | 26.3 ± 14.6 | | 0.046^※^ | 1 month | 9.3 ± 5.3 | | <0.01^※^ | |
| 3 months | 22.8 ± 15.7 | | <0.01^※^ | 3 months | 8.3 ± 5.5 | | <0.01^※^ | |
| 6 months | 22.6 ± 16.4 | | <0.01^※^ | 6 months | 8.5 ± 5.6 | | <0.01^※^ | |
| 12 months | 22.6 ± 17.5 | | <0.01^※^ | 12 months | 8.2 ± 6.1 | | <0.01^※^ | |
| Stiffness subscale | **Mean value ± S.D.** | | **P value** | **Physical function subscale** | | **Mean value ± S.D.** | | **P value** |
| Preoperative | 3.4 ± 1.9 | |  | Preoperative | | 20.6 ± 12.6 | |  |
| 1 month | 2.7 ± 1.8 | | 0.07 | 1 month | | 17.0 ± 10.3 | | 0.14 |
| 3 months | 2.6 ± 1.9 | | 0.046^※^ | 3 months | | 14.6 ± 11.4 | | 0.02^※^ |
| 6 months | 2.4 ± 1.8 | | <0.01^※^ | 6 months | | 14.1 ± 11.6 | | <0.01^※^ |
| 12 months | 2.6 ± 2.0 | | 0.049^※^ | 12 months | | 14.4 ± 12.3 | | 0.01^※^ |
| Visual analog scale | | | | | | | | |
|  | | **Mean value ± S.D.** | **P value** |  |  | |  | |
| Preoperative | | 46.5 ± 23.5 |  |  |  | |  | |
| 1 month | | 30.1 ± 18.8 | <0.01^※^ |  |  | |  | |
| 3 months | | 27.3 ± 17.6 | <0.01^※^ |  |  | |  | |
| 6 months | | 27.4 ± 18.8 | <0.01^※^ |  |  | |  | |
| 12 months | | 32.8 ± 24.7 | <0.01^※^ |  |  | |  | |
| Japanese Knee Osteoarthritis Measure | | | | | | | | |
| Total Score | | **Mean value ± S.D.** | **P value** | **Pain and stiffness in knees** | **Mean value ± S.D.** | | **P value** | |
| Preoperative | | 34.9 ± 18.2 |  | Preoperative | 14.8 ± 7.4 | |  | |
| 1 month | | 30.5 ± 17.1 | 0.26 | 1 month | 10.5 ± 5.5 | | <0.01^※^ | |
| 3 months | | 25.8 ± 17.6 | 0.02^※^ | 3 months | 9.6 ± 5.4 | | <0.01^※^ | |
| 6 months | | 24.5 ± 17.8 | <0.01^※^ | 6 months | 9.3 ± 5.9 | | <0.01^※^ | |
| 12 months | | 26.8 ± 19.7 | 0.04^※^ | 12 months | 10.4 ± 6.5 | | <0.01^※^ | |
| Condition in daily life | | **Mean value ± S.D.** | **P value** | **General activities** | **Mean value ± S.D.** | | **P value** | |
| Preoperative | | 11.8 ± 7.6 |  | Preoperative | 6.2 ± 5.0 | |  | |
| 1 month | | 9.9 ± 6.7 | 0.18 | 1 month | 8.0 ± 5.9 | | 0.10 | |
| 3 months | | 9.0 ± 7.5 | 0.07 | 3 months | 4.8 ± 4.8 | | 0.17 | |
| 6 months | | 8.2 ± 7.2 | 0.02^※^ | 6 months | 4.9 ± 4.7 | | 0.23 | |
| 12 months | | 9.1 ± 8.1 | 0.09 | 12 months | 5.0 ± 4.9 | | 0.27 | |
| Health conditions | | **Mean value ± S.D.** | **P value** |  |  | |  | |
| Preoperative | | 3.1 ± 3.0 |  |  |  | |  | |
| 1 month | | 2.4 ± 1.6 | 0.08 |  |  | |  | |
| 3 months | | 2.3 ± 1.6 | 0.049^※^ |  |  | |  | |
| 6 months | | 2.0 ± 1.5 | 0.01^※^ |  |  | |  | |
| 12 months | | 2.3 ± 1.6 | 0.06 |  |  | |  | |
| Knee injury and Osteoarthritis Outcome Score | | | | | | | | |
| Average Score of 5 subscales | | **Mean value ± S.D.** | **P value** | **Pain subscale** | **Mean value ± S.D.** | | **P value** | |
| Preoperative | | 48.7 ± 15.8 |  | Preoperative | 53.1 ± 16.4 | |  | |
| 1 month | | 55.2 ± 17.6 | 0.04^※^ | 1 month | 62.0 ± 17.9 | | <0.01^※^ | |
| 3 months | | 58.6 ± 15.4 | <0.01^※^ | 3 months | 66.5 ± 16.6 | | <0.01^※^ | |
| 6 months | | 59.2 ± 15.8 | <0.01^※^ | 6 months | 66.0 ± 16.3 | | <0.01^※^ | |
| 12 months | | 58.6 ± 16.8 | <0.01^※^ | 12 months | 66.3 ± 15.6 | | <0.01^※^ | |
| Symptoms subscale | | **Mean value ± S.D.** | **P value** | **Activity of daily living subscale** | **Mean value ± S.D.** | | **P value** | |
| Preoperative | | 57.8 ± 18.4 |  | Preoperative | 70.0 ± 16.2 | |  | |
| 1 month | | 63.7 ± 19.3 | 0.11 | 1 month | 74.8 ± 16.0 | | 0.12 | |
| 3 months | | 67.4 ± 16.8 | <0.01^※^ | 3 months | 77.5 ± 16.1 | | 0.02^※^ | |
| 6 months | | 67.9 ± 17.0 | <0.01^※^ | 6 months | 79.3 ± 13.7 | | <0.01^※^ | |
| 12 months | | 67.0 ± 19.1 | 0.01^※^ | 12 months | 77.5 ± 15.6 | | 0.02^※^ | |
| Sports subscale | | **Mean value ± S.D.** | **P value** | **Quality of life subscale** | **Mean value ± S.D.** | | **P value** | |
| Preoperative | | 27.6 ± 19.0 |  | Preoperative | 33.6 ± 20.6 | |  | |
| 1 month | | 34.9 ± 24.8 | 0.18 | 1 month | 40.6 ± 21.3 | | 0.11 | |
| 3 months | | 37.1 ± 23.4 | 0.04^※^ | 3 months | 44.9 ± 21.1 | | 0.01^※^ | |
| 6 months | | 37.8 ± 23.3 | 0.03^※^ | 6 months | 45.1 ± 21.9 | | <0.01^※^ | |
| 12 months | | 37.5 ± 24.3 | 0.03^※^ | 12 months | 44.6 ± 24.6 | | 0.01^※^ | |
| ^※^ Statistically significant  Standard deviation (S.D.) | | | | | | | | |
